# The Highest Oxidation State of Rhodium: Rhodium(VII) in [RhO_3_]^+^

**DOI:** 10.1002/anie.202207688

**Published:** 2022-08-10

**Authors:** Mayara da Silva Santos, Tony Stüker, Max Flach, Olesya S. Ablyasova, Martin Timm, Bernd von Issendorff, Konstantin Hirsch, Vicente Zamudio‐Bayer, Sebastian Riedel, J. Tobias Lau

**Affiliations:** ^1^ Physikalisches Institut Albert-Ludwigs-Universität Freiburg Hermann-Herder-Straße 3 79104 Freiburg Germany; ^2^ Abteilung für Hochempfindliche Röntgenspektroskopie Helmholtz-Zentrum Berlin für Materialien und Energie Albert-Einstein-Straße 15 12489 Berlin Germany; ^3^ Institut für Chemie und Biochemie–Anorganische Chemie Freie Universität Berlin Fabeckstraße 34/36 14195 Berlin Germany

**Keywords:** Gas Phase, Oxidation State, Oxides, Rhodium, X-Ray Absorption Spectroscopy

## Abstract

Although the highest possible oxidation states of all transition elements are rare, they are not only of fundamental interest but also relevant as potentially strong oxidizing agents. In general, the highest oxidation states are found in the electron‐rich late transition elements of groups 7–9 of the periodic table. Rhodium is the first element of the 4d transition metal series for which the highest known oxidation state does not equal its group number of 9, but reaches only a significantly lower value of +6 in exceptional cases. Higher oxidation states of rhodium have remained elusive so far. In a combined mass spectrometry, X‐ray absorption spectroscopy, and quantum‐chemical study of gas‐phase$\;{\left[{RhO}_{n}\right]}^{+}$ (*n*=1–4), we identify ${\left[{RhO}_{3}\right]}^{+}$ as the ${}^{1}A_{1}{}^{'}$ trioxidorhodium(VII) cation, the first chemical species to contain rhodium in the +7 oxidation state, which is the third‐highest oxidation state experimentally verified among all elements in the periodic table.

## Graphical abstract

Teaming up with technetium and ruthenium, rhodium is the third element of the 4d series to form the rare +7 oxidation state, the third‐highest oxidation state among all elements. Here, gas‐phase X‐ray absorption spectroscopy, combined with quantum‐chemical calculations, are used to elucidate ${\left[{\mathrm{RhO}}_{3}\right]}^{+}$ as a ${}^{1}A_{1}{}^{'}$ trioxido rhodium(VII) cation in $D_{3h}$ point group symmetry, the first chemical species with rhodium in the +7 oxidation state.

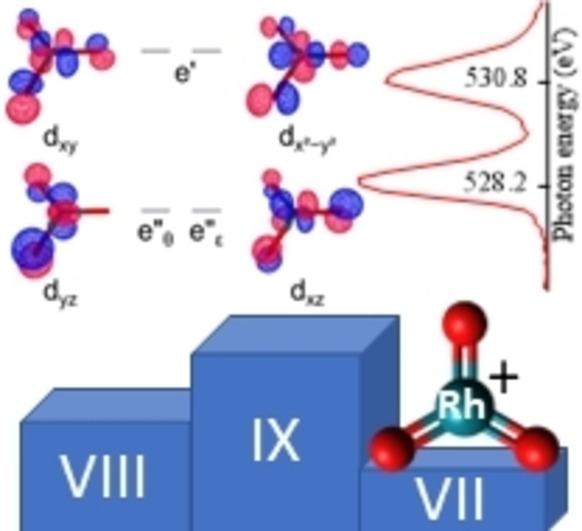

The formal concept of oxidation states is of fundamental importance in chemistry to characterize properties of elements in compounds.[^1^, ^2^] The search for compounds with transition metals in their highest oxidation states[^1^, ^3^, ^4^, ^5^, ^6^] is not only of academic interest, but complexes with metal centers in unusually high oxidation states are of particular relevance in the context of strong oxidants[^7^, ^8^, ^9^] or fluorinating agents,[^10^, ^11^, ^12^, ^13^] in catalytic processes,[^14^, ^15^, ^16^, ^17^, ^18^, ^19^] and as intermediates in key reactions.[^20^, ^21^]

Of the 4*d* transition elements, ruthenium, in the form of tetraoxido ruthenium(VIII),^[22]^ is the last element for which the highest experimentally verified oxidation state corresponds to its group number. The highest known oxidation state for the next element in this series, rhodium, is +6 as has been proven for rhodium(VI) hexafluoride and a few other compounds.^[23]^ In contrast, the highest oxidation states of its heavier 5d congener, iridium, agrees with its group number, and the tetraoxido iridium(IX) cation, ${[\mathrm{IrO}}_{4}{]}^{+}$ , represents the only known species with an element in oxidation state +9.^[24]^ An even higher oxidation state has been claimed for group 10 with platinum(X) in [PtO_4_]^2+^,^[6]^ although such species have not been verified experimentally so far, in line with the prediction that the dicationic species would decay into ${[\mathrm{PtO}}_{2}{]}^{+}$ .^[5]^ Among the tetraoxido cations, the stability of group 9 elements in high oxidation states is compromised for the lighter congeners, cobalt and rhodium, because of the decreasing number of radial nodes in the 4d and 3d orbitals, which leads to less effective overlap with ligand orbitals.[^1^, ^4^] Such behavior is assigned mainly to Pauli repulsion between ligand‐based orbitals as the contraction of the metal *d* orbitals increases for higher oxidation states.[^1^, ^4^] While nonavalent rhodium has been predicted in [RhO_4_]^+^ and RhNO_3_, it has also been pointed out that highly covalent bonds in these species might lead to reconfiguration into isomers with rhodium in a lower oxidation state,^[25]^ or even to the decay of ${\left[{\mathrm{RhO}}_{4}\right]}^{+}$ by oxygen elimination.^[26]^ Consequently, experimental and theoretical investigations show that rhodium forms dioxido‐superoxido $\left[\left(\eta^{1}-O_{2}\right){\mathrm{RhO}}_{2}\right]$ or dioxido‐peroxido $\left[\left(\eta^{2}-O_{2}\right){\mathrm{RhO}}_{2}\right]$ species with oxidation states of +5 and +6 instead of the tetraoxido isomer, ${\mathrm{RhO}}_{4}$ ,[^27^, ^28^] but the formation of ${\left[{\mathrm{RhO}}_{3}\right]}^{+}$ , as a candidate for rhodium(VII), has not been investigated experimentally or computationally so far.

Here, we present a gas‐phase mass spectrometry and X‐ray absorption spectroscopy study, combined with quantum‐chemical calculations, of ${\left[{\mathrm{RhO}}_{n}\right]}^{+}$ (*n*=0–4) cations. The formal oxidation states of rhodium in ${\left[{\mathrm{RhO}}_{n}\right]}^{+}$ (*n*=0–4) are identified by rhodium $M_{3}$ and oxygen K‐edge X‐ray absorption spectroscopy with local excitation at the rhodium center or at the oxygen ligands, respectively, giving independent access to the local, non‐bonding 4d configuration of rhodium, and to the nature of the oxygen species. For ${\left[{\mathrm{RhO}}_{3}\right]}^{+}$ we also compare the experimental data to the simulated oxygen K‐edge spectrum of the lowest energy isomer. Our results identify ${\left[{\mathrm{RhO}}_{3}\right]}^{+}$ as a trioxido rhodium(VII) cation, the first chemical species containing rhodium in the formal +7 oxidation state.

The experiments were performed at the ion‐trap endstation, located at beamline UE52‐PGM of the BESSY II synchrotron radiation facility, and computations were carried out using density functional theory as well as multireference methods. The experimental setup is described elsewhere,^[29]^ and details of the experimental and computational methods are given in the Supporting Information.

In Figure 1, the experimental X‐ray absorption spectrum of ${\left[{\mathrm{RhO}}_{3}\right]}^{+}$ at the oxygen K‐edge is shown, which is characterized by two main lines at 528.2 and 530.8 eV. These main features agree well with the X‐ray absorption spectrum simulated with time‐dependent density functional theory methods (TD‐DFT, see Supporting Information for details), also shown in Figure 1, of ${\left[{\mathrm{RhO}}_{3}\right]}^{+}$ in the ${}^{1}A_{1}{}^{'}$ electronic ground state in $D_{3h}$ point group symmetry. The calculated full spectrum is shown in Figure S6, where we see a poorer agreement between experiment and theory for excitations of higher energy at the oxygen K‐edge. This effect is expected for TD‐DFT studies as a consequence of the approximations made.^[30]^ In order to compare absolute energies with the experimental data, the calculated spectrum was shifted by +19.7 eV. The structure optimizations of ${\left[{\mathrm{RhO}}_{3}\right]}^{+}$ converge to a trigonal planar structure of *D*_3*h*_ symmetry with bond lengths of 167–169 pm, see Table S6, which are comparable with the bond length expected for a rhodium–oxygen double bond based on additive covalent radii of 167 pm.^[31]^

**Figure 1:**
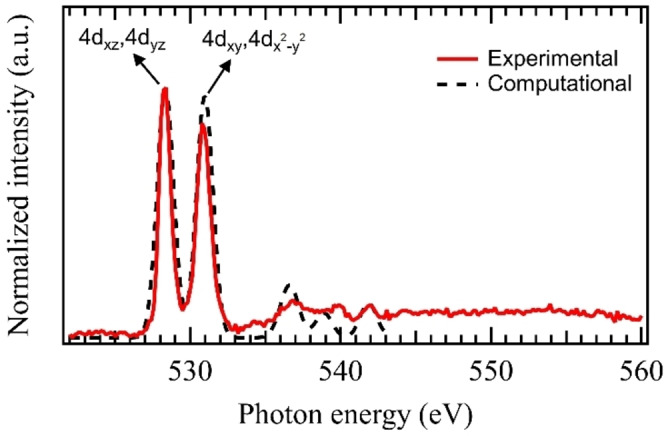
Experimental ion yield spectrum (solid red line) and computational TD‐DFT X‐ray absorption spectrum for the ${}^{1}A_{1}{}^{'}$ ground state (dashed black line) of ${\left[{\mathrm{RhO}}_{3}\right]}^{+}$ , at the oxygen K‐edge, with very good agreement for the transitions below 542 eV.

Figure 2 shows a depiction of the ${}^{1}A_{1}{}^{'}$ electronic ground state of ${\left[{\mathrm{RhO}}_{3}\right]}^{+}$ , where the valence electrons are paired in the non‐bonding *a*_2_′′ and *a*_1_′ molecular orbitals, representing the oxygen‐centered 2p_*z*_ and rhodium‐centered $4d_{z^{2}}$ atomic orbitals, respectively. The two‐electron occupation of the *a*_1_′ orbital, along with the low electron density at any other d‐derived molecular orbitals, suggest rhodium has a local 4d^2^ electronic configuration, which is consistent with an oxidation state of +7. Both components of the twofold degenerate *e*′′ orbital show out‐of‐plane interaction of the atomic orbitals along the bonds of anti‐bonding π* character, and are represented by *e*′′_*θ*_ and *e*′′_*∈*_, for main rhodium atomic orbital contributions, d_*yz*_ and d_*xz*_, respectively, while the components of the *e*′ orbital show in‐plane interactions, also of antibonding π* character.

**Figure 2:**
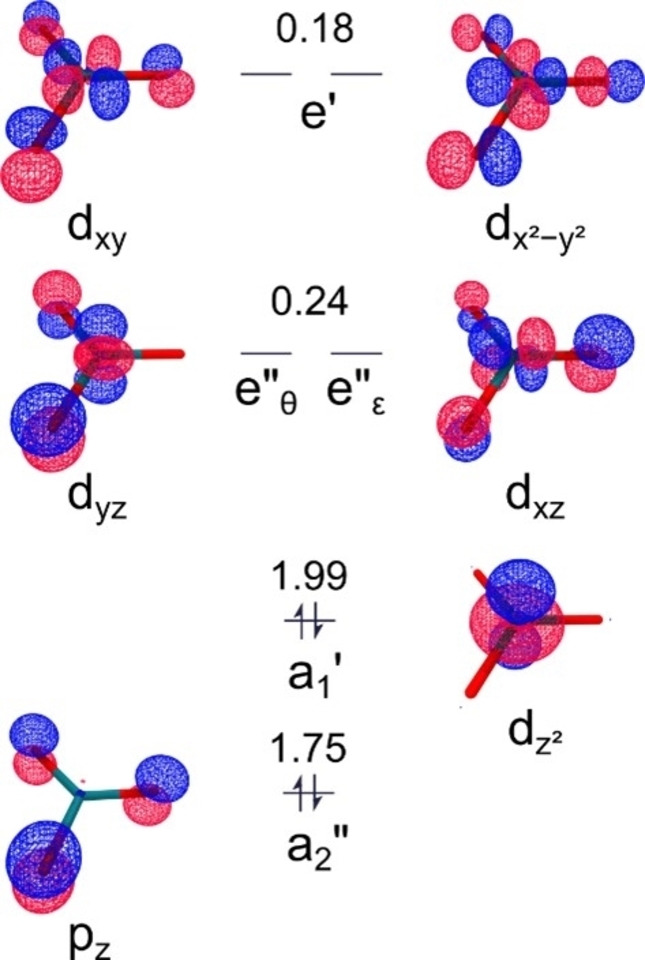
Frontier natural molecular orbital plot at 0.1 e Bohr^−3^ (state‐specific CASSCF(15,20)/aug‐cc‐pVTZ‐DK) for the ${}^{1}A_{1}{}^{'}$ electronic ground state in *D*_3*h*_ point group symmetry of ${\left[{\mathrm{RhO}}_{3}\right]}^{+}$ spanning the rhodium valence d orbital space, *a*_1_′, *e*′′ and *e*′, as well as the oxygen ligand centered *a*_1_′′ orbital. Arrows indicate the electron distribution of the leading configuration, while fractional numbers show the natural occupations. The fully (1.99 electrons) occupied, non‐bonding *a*_1_′ (4d^2^) orbital indicates rhodium in the formal +7 oxidation state.

As indicated in Figure 1, the main features at the oxygen K‐edge correspond to electronic excitations from the oxygen 1s orbital to the molecular *e*′′ and *e*′ orbitals, in which the main rhodium atomic orbital contributions are 4d_*xz*_ and 4d_*yz*_, for the 528.2 eV line, and 4d_*xy*_ and $4d_{x^{2}-y^{2}}$ , for the 530.8 eV transition. This is in line with the very similar oxygen K‐edge X‐ray absorption spectrum of gas‐phase ${}^{1}A_{1}{}^{'}\;{\left[{\mathrm{MnO}}_{3}\right]}^{+}$ , where two strong lines at 528.2 and 530.8 eV are also observed for the trioxido manganese(VII) cation.^[32]^

We have also investigated the lowest four excited states of ${\left[{\mathrm{RhO}}_{3}\right]}^{+}$ , see Supporting Information, the first two of which are triplet states, representing partial oxidations of the oxido ligands, and are energetically well separated from the electronic ground state by about 50 kJ mol^−1^, see Table S2. The X‐ray absorption spectrum at the oxygen K‐edge was also calculated for these triplet states of ${\left[{\mathrm{RhO}}_{3}\right]}^{+}$ , but the band separations as well as the intensity ratios for the π* transitions are not consistent with the experimental data, cf. Figure S6.

An overview of the oxygen K‐edge spectra of the complete ${\left[{\mathrm{RhO}}_{n}\right]}^{+}$ (*n*=0–4) series is shown in Figure 3. Here, the spectrum of ${\left[\mathrm{RhO}\right]}^{+}$ can be taken as a reference for the oxido ligand signature in the predicted ${}^{3}\Sigma^{-}$ ground state[^33^, ^34^] of ${\left[\mathrm{RhO}\right]}^{+}$ with a bond dissociation energy of 3 eV.^[35]^ The electronic configuration of ${\left[\mathrm{RhO}\right]}^{+}$ suggests that the line observed at 528.72 eV corresponds to a transition into the oxygen $1s^{-1}6\pi^{3}$ excited state. Since the very similar spectrum of ${\left[{\mathrm{RhO}}_{2}\right]}^{+}$ also indicates oxido ligands, in line with neutral $\left[{\mathrm{RhO}}_{2}\right]$ species,^[27]^ only oxido ligands are identified for the ${\left[{\mathrm{RhO}}_{n}\right]}^{+}$ (*n*=1–3) series, and the formal oxidation states of rhodium can be assigned as +3, +5, and +7, respectively. In contrast, the ${\left[{\mathrm{RhO}}_{4}\right]}^{+}$ species shows a $\sigma^{{}^{*}}$ ‐like transition around 540 eV, similar to the $1\sigma_{g}\to 3\sigma_{u}^{{}^{*}}$ transition of molecular oxygen.[^36^, ^37^] This $\sigma^{{}^{*}}$ resonance is an indication of the existence of an oxygen–oxygen bond, and is a characteristic spectroscopic fingerprint of chemisorbed or physisorbed dioxygen at metal surfaces in the peroxide or superoxide forms,[^38^, ^39^] of solid‐phase superoxides,[^40^, ^41^] and of peroxide units in $H_{2}O_{2}$ and organic compounds.[^42^, ^43^] This suggests the presence of at least one dioxygen unit in ${\left[{\mathrm{RhO}}_{4}\right]}^{+}$ , and thus confirms that tetraoxido rhodium is not formed.[^25^, ^27^, ^28^] Although the oxidation state of rhodium in ${\left[{\mathrm{RhO}}_{4}\right]}^{+}$ cannot be unambiguously assigned from the oxygen K‐edge alone, we tentatively assign the formal oxidation states of +4 or +5, see Supporting Information for details.

**Figure 3:**
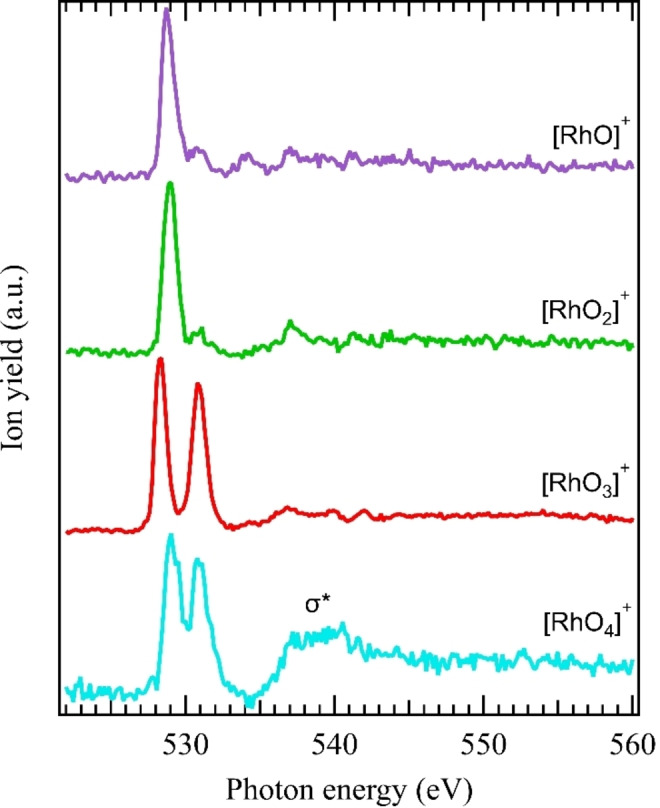
Ion yield spectra at the oxygen K edge of the ${\left[{\mathrm{RhO}}_{n}\right]}^{+}$ (*n*=0–4) series. The absence of any oxygen‐oxygen $\sigma^{{}^{*}}\;$ resonance for *n*=1–3 indicates purely oxido rhodium cations, while the presence of an oxygen‐oxygen $\sigma^{{}^{*}}$ resonance for ${\left[{\mathrm{RhO}}_{4}\right]}^{+}$ indicates the presence of at least one oxygen–oxygen bond.

To further corroborate the unusual +7 oxidation state of rhodium, we have determined the oxidation state of the rhodium center in ${\left[{\mathrm{RhO}}_{n}\right]}^{+}$ by evaluating chemical shifts of the rhodium $M_{3}$ excitation. This is a standard technique for 3d transition metals, which show a linear blueshift at the L edge as the oxidation state of the metal increases.[^30^, ^44^] A similar chemical shift has also been observed for 4d transition metals, where the $M_{3}$ edge of molybdenum shows a shift of 0.49 eV per unit of oxidation state.^[45]^

Rhodium $M_{3}$ ‐edge X‐ray absorption spectra were measured for ${\left[{\mathrm{RhO}}_{n}\right]}^{+}$ (*n*=0–4), see Supporting Information. The median $M_{3}$ excitation energy, calculated from the integrated intensity of the ${\left[{\mathrm{RhO}}_{n}\right]}^{+}$ (*n*=0–4) spectra, cf. Table 1, indeed shows a systematic blueshift when plotted as a function of the formal oxidation state of the rhodium center in each species, cf. Figure 4, where ${\left[{\mathrm{RhO}}_{4}\right]}^{+}$ is omitted because the oxidation state was not determined unambiguously. This correlation can be fitted linearly as $E_{M_{3}}=a+b\cdot OS_{Rh}$ , where $E_{M_{3}}$ is the median of the rhodium $M_{3}$ excitation energy, $OS_{Rh}$ is the formal oxidation state of the rhodium atom, with coefficients *a*=493.85±0.19 eV and *b*=0.88±0.04 eV. In Table 1 and Figure 4 we have also included the formal occupation numbers of the non‐bonding rhodium 4d derived valence orbitals, which are related to the rhodium $M_{3}$ ‐edge shift and oxidation states,[^2^, ^44^] for the rhodium cation and oxido rhodium cation series. We observe a chemical shift of the median $M_{3}$ ‐edge excitation energy of 0.88±0.04 eV per unit of oxidation state, which is comparable to the chemical shift of 0.82 eV observed at the $L_{3}$ ‐edge of iron^[46]^ and, in general, is between the values found for the molybdenum $M_{3}$ ‐edge, of 0.49 eV,^[45]^ and for the $L_{3}$ ‐edge of 3d transition metals, of 1–2 eV.[^46^, ^47^] The observed linear dependence of $M_{3}$ shift and rhodium oxidation state is a confirmation of the assigned +7 oxidation state in ${\left[{\mathrm{RhO}}_{3}\right]}^{+}$ , in full agreement with our theoretical results and with the identification of a trioxido species from oxygen K‐edge X‐ray absorption spectroscopy.

**Table 1:** Median values of the $M_{3}$ ‐edge excitation energy, formal oxidation state (OS) of the rhodium atom and formal occupation of the rhodium‐derived 4d valence orbitals for rhodium cation and ${\left[{\mathrm{RhO}}_{n}\right]}^{+}$ (*n*=1–3) oxido species.

	Formal OS of Rh	Rh(4d) formal occupation	Median [eV]
${\mathrm{Rh}}^{+}$	+1	4d^8^	494.67±0.15
${\left[\mathrm{RhO}\right]}^{+}$	+3	4d^6^	496.48±0.15
${\left[{\mathrm{RhO}}_{2}\right]}^{+}$	+5	4d^4^	498.49±0.15
${\left[{\mathrm{RhO}}_{3}\right]}^{+}$	+7	4d^2^	499.90±0.15

**Figure 4:**
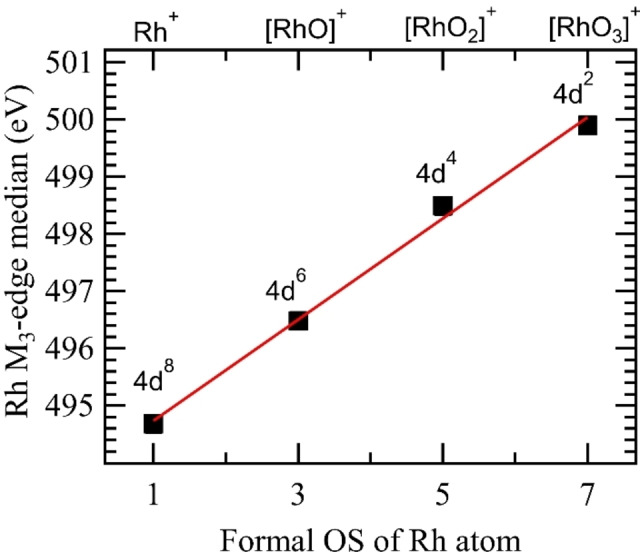
Median values, calculated from the integrated intensity of the rhodium $M_{3}$ edge of ${\left[{\mathrm{RhO}}_{n}\right]}^{+}$ (*n*=0–3) species, plotted as a function of the formal oxidation state of the rhodium center, with a linear fit shown as a red line. The formal occupation of the (non‐bonding) rhodium 4d atomic orbitals is indicated in the figure. Cf. Table 1 for numerical values.

In summary, we have characterized the highest oxidation state of rhodium observed so far, rhodium(VII) in ${\left[{\mathrm{RhO}}_{3}\right]}^{+}$ , by combination of mass spectrometry, X‐ray absorption spectroscopy, and quantum‐chemical calculations. This makes rhodium the third 4d transition element to form the rare formal +7 oxidation state, along with technetium and ruthenium. Because of its unusually high oxidation state, ${\left[{\mathrm{RhO}}_{3}\right]}^{+}$ should be a strong oxidizing agent, and we would expect gas‐phase reactivity studies^[48]^ to reflect on this behavior. Our predicted vertical electron affinity of ${\left[{\mathrm{RhO}}_{3}\right]}^{+}$ , which is 10.6 eV and 11.1 eV at the CCSD(T) and B3LYP levels, respectively, is higher than 9.59 eV[^49^, ^50^] for NO_2_^+^ but significantly lower than 12.07 eV^[51]^ for O_2_^+^. Since O_2_^+^ is stabilized by weakly coordinating anions like [PtF_6_]^−^, [BF_4_]^−^, or [AsF_6_]^−^, it might be possible to also stabilize ${\left[{\mathrm{RhO}}_{3}\right]}^{+}$ , based on its electron affinity as shown, e.g., for the stabilization of [IrO_4_]^+^.^[26]^

## Conflict of interest

The authors declare no conflict of interest.

## Supporting information

As a service to our authors and readers, this journal provides supporting information supplied by the authors. Such materials are peer reviewed and may be re‐organized for online delivery, but are not copy‐edited or typeset. Technical support issues arising from supporting information (other than missing files) should be addressed to the authors.

Supporting Information

## Data Availability

The data that support the findings of this study are available from the corresponding author upon reasonable request.
