# Supplementary material for: The Highest Oxidation State of Rhodium: Rhodium(VII) in [RhO3]+
Source: Angew Chem Int Ed Engl. 2022 Aug 10;61(38):e202207688. doi: 10.1002/anie.202207688 (PMC9544489; doi:10.1002/anie.202207688)
Supplement: Supplementary file 1 — Supporting Information [file ANIE-61-0-s001.pdf]

Angewandte Chemie
Eine Zeitschrift der Gesellschaft Deutscher Chemiker

Supporting Information

The Highest Oxidation State of Rhodium: Rhodium(VII) in $[RhO_3]^+$

M. da Silva Santos, T. Stüker, M. Flach, O. S. Ablyasova, M. Timm, B. von Issendorff, K. Hirsch, V. Zamudio-Bayer, S. Riedel*, J. T. Lau**

1 Experimental details

$[RhO_n]^+$ ($n = 0 - 3$) molecular ions were produced by argon sputtering of a rhodium target in the presence of oxygen, which was introduced as a mixture of 1% oxygen in helium carrier gas. In the plasma region of the ion source, ozone is formed[1] that reacts to produce cationic, anionic and neutral rhodium-oxygen species of different sizes and compositions.[2–4] The magnetron sputter source was kept at room temperature.

The cationic species are directed via electrostatic fields and a radio frequency ion guide to a radio frequency quadrupole mass filter, where the ions of interest are selected. The mass selected ions are guided into a linear radio frequency quadrupole ion trap, which is cooled by liquid helium. The ion trap has a pulsed exit aperture where the stored ions are extracted in bunches, and are thereafter mass analyzed by a reflectron time-of-flight mass spectrometer. Fig. S1 shows the mass spectra of the investigatred species, $[RhO_n]^+$ ($n = 0 - 3$).

The ion trap is aligned with the beamline, allowing the interaction of the X-rays with the stored ions. X-ray absorption by the ions is followed by multiple Auger decay leading to the dissociation of the ions due to Coulomb repulsion. The ion yield spectrum is obtained by monitoring the product ion intensity with the time-of-flight mass spectrometer, while scanning the photon energy over an absorption edge. The incident photon energy was scanned in steps of 160 meV with a photon energy bandwidth of 345 meV at the oxygen K-edge and steps of 150 meV with a photon energy bandwidth of 300 meV at the rhodium M_3-edge.

The oxygen K-edge spectra obtained from different ion yield channels are shown in Fig. S2. The most intense product ions, Rh^{2+} and O^+ for samples $[RhO_{1,2}]^+$, and Rh^+ and O^+ for $[RhO_{3,4}]^+$, resulted in very similar ion yield spectra for a respective parent ion, indicating that the partial ion yield spectra are a good approximation of the total ion yield, and thus to the X-ray absorption spectrum. The figure in the main text uses the ion yield channel that showed the highest signal-to-noise ratio, which is the O^+ yield in all cases.

For the rhodium M_3-edge plots, only the Rh^{2+} product ion was observed with significant intensity, therefore, all plots shown are from the Rh^{2+} yield channel. All ion yield spectra showed in this work are normalized to a 0–1 intensity range.

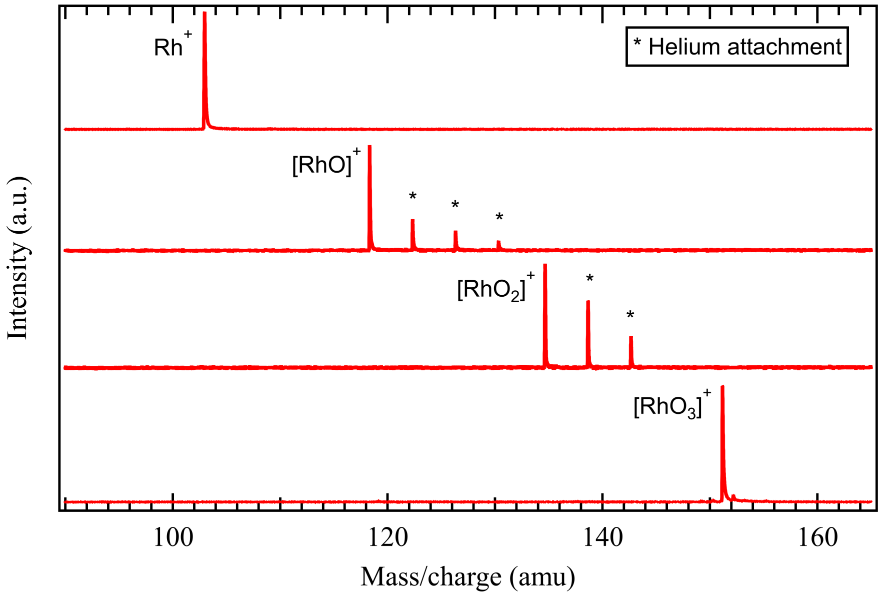

Figure S1: TOF mass spectra of trapped, mass-selected ions $[RhO_n]^+$ ($n = 0 - 3$).

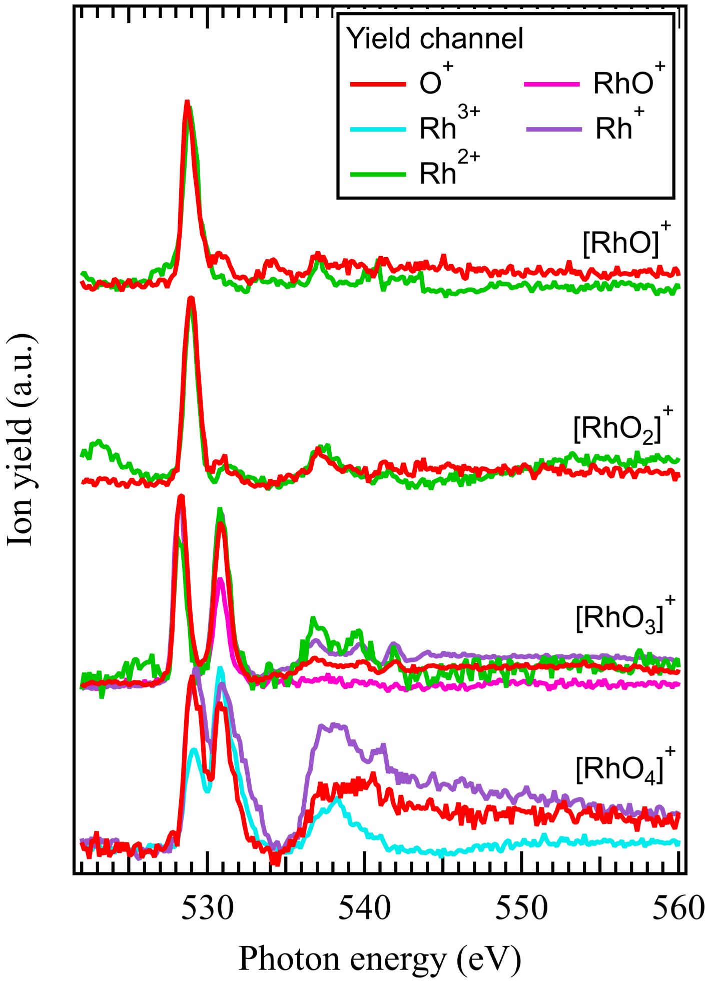

Figure S2: Oxygen K-edge spectra from different ion yield channels of the $[RhO_n]^+$ ($n = 1 - 4$) series.

2 Rhodium M_3-edge spectra

Fig. S3 shows the rhodium M_3-edge spectra of the cationic rhodium-oxo series $[RhO_n]^+$ ($n = 0 - 3$), indicating the chemical shift on the position of the peak median along the series as the oxidation state (OS) of the rhodium atom increases.

In order to minimize the experimental uncertainty, only the energy range that comprises the absorption peak was considered to calculate the median (Table S1). We plot the integral value for this region, done with the raw data, as a function of the energy. We then extract the energy position of the point at half of the height of the integral curve. This process was done individually for two scans of each sample and the average value of the median was considered.

Table S1: Energy range considered to calculate the median of Rh M_3-edge.

	Energy range (eV)
Rh^+	488.00 – 501.56 ± 0.15
$[RhO]^+$	488.00 – 501.56 ± 0.15
$[RhO_2]^+$	490.04 – 503.48 ± 0.15
$[RhO_3]^+$	489.95 – 504.95 ± 0.15

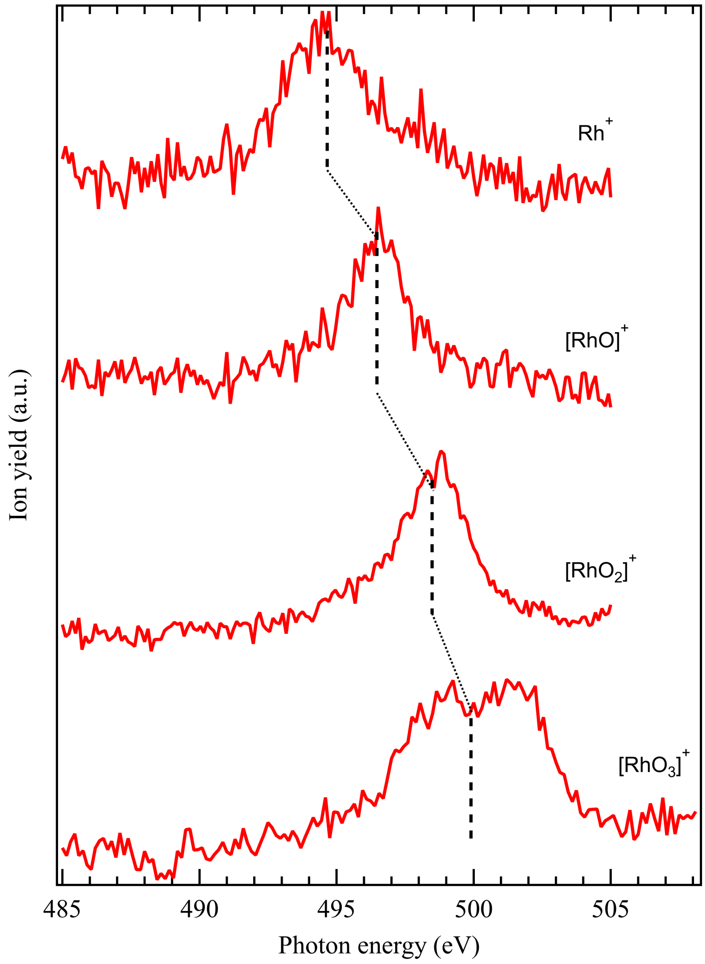

Figure S3: Ion yield spectra at the rhodium M_3-edge of the rhodium oxides series $[RhO_n]^+$ ($n = 0 - 3$). The Rh^{2+} yield channel is shown here for all spectra. The black dashed lines indicate the position of the peak median.

3 The case of $[RhO_4]^+$

In this study, also the $[RhO_4]^+$ species was observed, although at much lower intensity, as evidenced by the smaller signal-to-noise ratio when comparing the mass spectrum of the selected $[RhO_4]^+$ (Fig. S4) with the other rhodium-oxo species in Fig. S1. The presence of a σ^*-like transition around 540 eV at the oxygen K-edge of $[RhO_4]^+$ in Fig. 3 suggests the presemce of an O_2 unit.[5] The rhodium M_3-edge spectrum is shown in Fig. S5. The peak median at the rhodium M_3-edge was calculated subtracting a linear background from the raw data and extracting the half of the peak integral. The position of the Rh M_3-edge median of $[RhO_4]^+$ is at 497.90 ± 0.15 eV, which when inserted in the equation of the linear fit showed in Fig. 3, predicts an OS $= 4.6 \pm 0.3$ for the Rh atom in $[RhO_4]^+$. Therefore, two possibilities arise, a cationic peroxo-superoxo system for OS(Rh) $= 4$, or a cationic diperoxo system for an OS(Rh) $= 5$, and a distinction could be made by a deeper theoretical study combined with these results. Though it is not clear how the oxygen atoms are bonded to the Rh atom in the $[RhO_4]^+$ system, it is evident that we do not have a cationic tetroxide system and the trioxide cation is the highest-oxidized rhodium-oxo system we could produce.

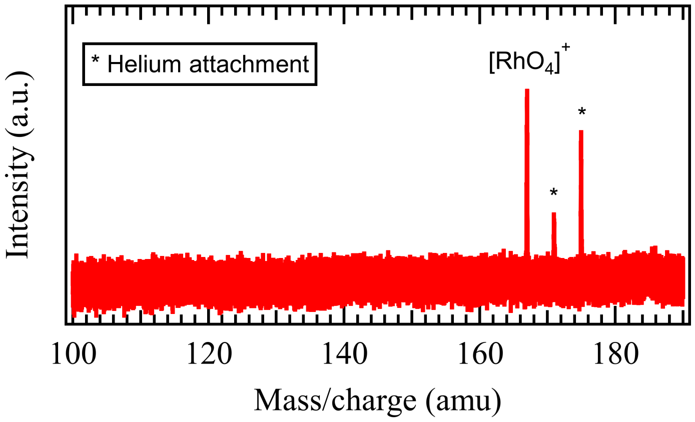

Figure S4: Mass spectrum of selected ions $[RhO_4]^+$.

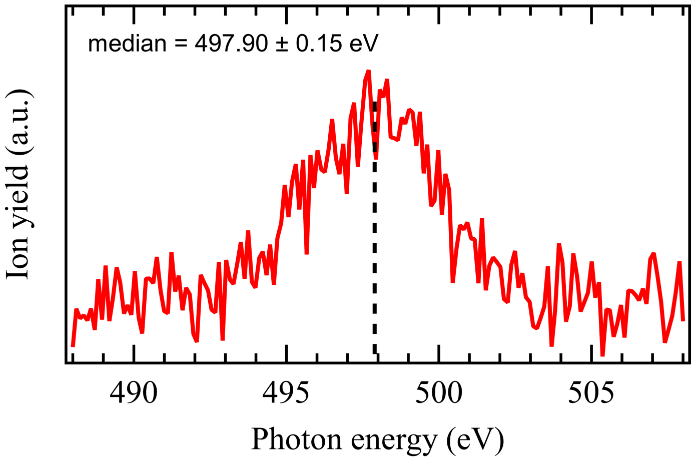

Figure S5: Ion yield spectrum of $[RhO_4]^+$ at the rhodium M_3-edge, where the dashed black line indicates the position of the peak median. The Rh^{2+} yield channel is shown here.

4 Computational results

The structures were optimized using spin-restricted (closed-shell species) and spin-unrestricted (open-shell species) density functional theory (DFT) methods, B3LYP [6–9] and M06-L,[10, 11] combined with the triple zeta def2-TZVP basis sets and the associated ECP [12–14] using TURBOMOLE 7.1[15], as well as at the CCSD(T)/aug-cc-pVTZ(-PP) level [16, 17] using Molpro 2019 [18–20]. The stability of the spin-restricted DFT ground state was verified by the positivity of the lowest eigenvalues in all IRREPs for B3LYP and M06L. The X-ray absorption spectrum was calculated using time-dependent density functional theory (TD-DFT) BP86 [8, 21] together with the ZORA-def2-TZVP [14] basis set for oxygen, SARC-ZORA-TZVP for rhodium, and the auxiliary basis SARC/J [22, 23] using ORCA 5.0.1 [24] with its default RI settings. The TD-DFT equations were solved using the Tamm–Dancoff approximation as implemented by default in ORCA 5.0.1,[25] with the slight modification that only excitations out of the localized oxygen 1s-orbital were allowed.

Scalar relativistic effects for this calculation were considered using the zeroth order regular expression (ZORA) as implemented in ORCA. The exact origin independent transition moments were obtained using the keywords “DoQuad”, “DoLength”, and “DoVelocity”.

To unequivocally determine the electronic ground state, single point calculations at the optimized structures were performed at the CASSCF(14,20), CASSCF(15,20), NEVPT2, CASPT2 and CCSD(T) levels of theory. The CASPT2 calculations were carried out without IPEA shift. To account for scalar relativistic effects, the DKH Hamiltonian was used together with the aug-cc-pVTZ-DK[16] basis sets for the multi-reference methods CASSCF, NEVTP2, and CASPT2. The 14-orbital active space spans the MOs formed by the 4d(Rh), as well as the 2p(O) atomic orbitals. The 15-orbital active space additionally includes the MO mainly formed by the 5s(Rh) atomic orbital.

Geometry optimizations at the DFT and CCSD(T) levels of theory converge to trigonal planar structures (D_{3h}) with bond lengths of 167 pm (B3LYP), 168 pm (M06L) and 169 pm (CCSD(T), see Table S5). The different electronic configurations arise by distributing the four lowest energy electrons in the a_2'', a_1', and e'' frontier molecular orbitals (MOs) in the D_{3h} high symmetry reference geometry shown in Figure 1. In the ${}^1A_1'$ ground state, S_0, the electrons are paired in the non-bonding a_1' and a_2'' MOs, $(a_2'')^2(a_1')^2(e'')^0$. The vertical electron affinity calculated for the ground state at the CCSD(T) level is 10.6 eV (B3LYP: 11.1 eV) The lowest excited state, $(a_2'')^2(a_1')^1(e'')^1(e')^0$, is prone to Jahn-Teller distortion and geometry relaxation leads to two triplet states of lower symmetries, C_{2v}–^{3}A$_2$ (T_1) and C_s–^{3}A$'$ (T_2), see Tables S6 and S7. Their corresponding electronic configurations are $(a_2'')^2(a_1')^1(e_\theta'')^1$ and $(a_2'')^2(a_1')^1(e_\epsilon'')^1$, respectively. The lowest ${}^5A_1''$ quintet state (Q_1) with an electron population of $(a_2'')^1(a_1')^1(e_\theta'')^1(e_\epsilon'')^1$ shows a highly symmetric D_{3h} point group symmetry. Lastly, the stationary point of ${}^3B_2$, the third triplet state (T_3) with an occupation pattern of $(a_2'')^2(a_1')^0(e_\theta'')^1(e_\epsilon'')^1$ exhibits C_{2v} symmetry, despite its totally symmetric charge distribution at the high symmetric reference geometry (Table S9). Vibrational analyses of the stationary points obtained at the DFT level were performed, see Tables S6–S10 that also lists the point groups in which the electronic structure calculations were performed.

The pre-edge region of the oxygen K-edge X-ray absorption spectrum of the S_0, T_1 and T_2 states shown in Fig. S6 was calculated using TD-DFT following the procedure outlined by Ray[26] at the BP86/ZORA-def2-TZVP(O)/SARC-ZORA-TZVP(Rh) level. Due to the degenerate nature of the oxygen 1s orbitals in D_{3h} point group symmetry 25 excitations from only one of the localized molecular orbitals corresponding to the oxygen

1s atomic orbitals into the entire virtual space were considered.

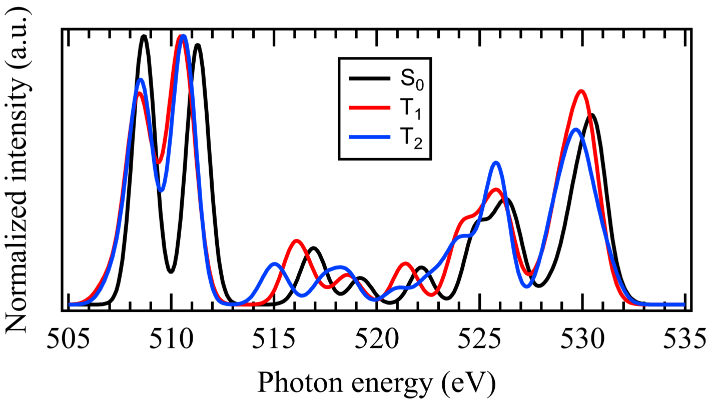

Figure S6: TD-DFT calculated X-ray absorption spectra at the oxygen K-edge for the S_0, T_1 and T_2 states of $[RhO_3]^+$.

The relative energies shown in Table S2 were calculated by using the lowest single point energy for the respective method at the given stationary point (Tables S3–S5). The singlet-triplet energy gap was predicted to be 17–75 kJ mol^{-1}. The almost identical energies gaps for both active spaces suggest that perturbative treatment of the CASSCF(14,20) recovers most of the correlation energy neglected by excluding the Rh(4s) orbital.

The CASPT2 method is suspected to systematically underestimate energies of open-shell states. [27, 28] A recent study has shown that this effect is minimal for organic chromophores using small basis sets, but often a stronger effect is still assumed for transition metal complexes in combination with larger basis sets. [29] Hence, the CCSD(T) and NEVPT2 energies of 46–64 kJ mol^{-1} can be considered a more reliable estimation of the singlet-triplet gap.

Table S2: Energy levels of the lowest electronic states. The enthalpy differences (ΔH, $T = 0$ K) were calculated from the electronic energies and the zero point energies.

State	$\Delta H_{\mathrm{CCSD(T)}}$	$\Delta H_{\mathrm{CASSCF(14)}^{a}}$	$\Delta H_{\mathrm{CASSCF(15)}^{a}}$	$\Delta H_{\mathrm{NEVPT2(14)}^{a}}$	$\Delta H_{\mathrm{CASPT2(14)}^{a}}$	$\Delta H_{\mathrm{CASPT2(15)}^{a}}$
S_0	0	0	0	0	0	0
T_1	67	61	75	46	18	17
T_2	64	60	75	47	22	21
Q_1	147	140	136	136	113	113
T_3	217	180	213	190	163	169

[a] The number indicates the number of active orbitals in the CASSCF reference function

Table S3: Single point energies at the optimized structures obtained at the B3LYP level. Electronic energies, E, in Hartree. The relative enthalpy at $T = 0$ K with respect to the lowest energy ($H = E + \mathrm{ZPE}_{\mathrm{B3LYP}}$) in kJ mol^{-1}.

	S_0	T_1	T_2	Q_1	T_3
E_{B3LYP} (ΔH)	-335.658222 (15)	-335.662280 (0)	-335.653436 (1)	-335.643283 (49)	-335.596945 (174)
$E_{\mathrm{CCSD(T)}}$ (ΔH)	-334.614266 (0)	-334.588170 (65)	-334.589457 (64)	-334.557437 (145)	-334.531778 (215)
$E_{\mathrm{CASSCF(14,20)}}$ (ΔH)	-5004.958955 (0)	-5004.936747 (54)	-5004.937825 (55)	-5004.906417 (133)	-5004.891432 (176)
$E_{\mathrm{NEVPT2(14,20)}}$ (ΔH)	-5005.789557 (0)	-5005.772890 (40)	-5005.773159 (42)	-5005.738227 (130)	-5005.718805 (184)
$E_{\mathrm{CASPT2(14,20)}}$ (ΔH)	-5005.849851 (0)	-5005.846074 (6)	-5005.844855 (12)	-5005.809427 (102)	-5005.790823 (153)
$E_{\mathrm{CASSCF(15,20)}}$ (ΔH)	-5004.995830 (0)	-5004.968538 (68)	-5004.969039 (70)	-5004.938918 (145)	-5004.916064 (208)
$E_{\mathrm{CASPT2(15,20)}}$ (ΔH)	-5005.848412 (0)	-5005.843908 (8)	–[a]	-5005.806632 (102)	-5005.786036 (162)

[a] CASPT2(15,20) omitted due to high computational cost in C_s point group symmetry.

Table S4: Single point energies at the optimized structures obtained at the M06L level. Electronic energies, E, in Hartree. The relative enthalpy at $T = 0$ K with respect to the lowest energy ($H = E + \text{ZPE}_{\text{M06L}}$) in kJ mol^{-1}.

	S_0	T_1	T_2	Q_1	T_3
E_{M06L} (ΔH)	-335.850841 (0)	-335.847309 (7)	-335.842265 (22)	-335.816094 (71)	-335.781630 (181)
$E_{\text{CCSD(T)}}$ (ΔH)	-334.615159 (0)	-334.588210 (68)	-334.589693 (66)	-334.557450 (147)	-334.531888 (218)
$E_{\text{CASSCF(14,20)}}$ (ΔH)	-5004.960110 (0)	-5004.936767 (59)	-5004.938137 (57)	-5004.906580 (136)	-5004.892106 (178)
$E_{\text{NEVPT2(14,20)}}$ (ΔH)	-5005.790690 (0)	-5005.773110 (43)	-5005.773396 (45)	-5005.738233 (134)	-5005.719038 (187)
$E_{\text{CASPT2(14,20)}}$ (ΔH)	-5005.851585 (0)	-5005.846099 (12)	-5005.844963 (17)	-5005.809533 (106)	-5005.791306 (158)
$E_{\text{CASSCF(15,20)}}$ (ΔH)	-5004.997061 (0)	-5004.968473 (72)	-5004.969379 (72)	-5004.939128 (148)	-5004.916765 (210)
$E_{\text{CASPT2(15,20)}}$ (ΔH)	-5005.849752 (0)	-5005.843943 (12)	–[a]	-5005.806732 (109)	-5005.786496 (169)

[a] CASPT2(15,20) omitted due to high computational cost in C_s point group symmetry.

Table S5: Single point energies at the optimized structures obtained at the CCSD(T) level. Electronic energies, E, in Hartree. The relative enthalpy at $T = 0$ K with respect to the lowest energy ($H = E + \text{ZPE}_{\text{CCSD(T)}}$) in kJ mol^{-}1.

	S_0	T_1	T_2	Q_1	T_3
$E_{\text{CCSD(T)}}$ (ΔH)	-334.616036 (0)	-334.588876 (67)	-334.592308 (62)	-334.557450 (149)	-334.531908 (219)
$E_{\text{CASSCF(14,20)}}$ (ΔH)	-5004.961634 (0)	-5004.934790 (67)	-5004.938418 (60)	-5004.906593 (140)	-5004.892467 (180)
$E_{\text{NEVPT2(14,20)}}$ (ΔH)	-5005.792044 (0)	-5005.771274 (51)	-5005.774069 (47)	-5005.738232 (137)	-5005.719131 (190)
$E_{\text{CASPT2(14,20)}}$ (ΔH)	-5005.854398 (0)	-5005.844560 (22)	-5005.845767 (22)	-5005.809541 (113)	-5005.791551 (163)
$E_{\text{CASSCF(15,20)}}$ (ΔH)	-5004.998787 (0)	-5004.966816 (80)	-5004.969786 (75)	-5004.939145 (152)	-5004.917143 (213)
$E_{\text{CASPT2(15,20)}}$ (ΔH)	-5005.851785 (0)	-5005.842363 (21)	-5005.843622 (21)[a]	-5005.806740 (114)	-5005.786728 (169)

[a] Converged to C_{2v} point group symmetry.

Table S6. Optimized structures, point groups used for calculations and vibrational data for the S_0 electronic state.

**B3LYP/def2-TZVP (D_{3h} - $^1A_1'$)**	
Cartesian coordinates	4<br>Energy = -335.6582219614<br>Rh -0.0000000 0.0000000 0.0000000<br>O 0.8339636 -1.4444674 0.0000000<br>O 0.8339636 1.4444674 0.0000000<br>O -1.6679273 0.0000000 0.0000000
Vibrational data	# mode symmetry wave number IR intensity selection rules<br># cm**(-1) km/mol IR RAMAN<br>7 a2" 113.24 6.71473 YES NO<br>8 e' 282.86 0.54286 YES YES<br>9 e' 282.86 0.54286 YES YES<br>10 e' 931.71 34.56424 YES YES<br>11 e' 931.71 34.56424 YES YES<br>12 a1' 957.71 0.00000 NO YES
**M06L/def2-TZVP (D_{3h} - $^1A_1'$)**	
Cartesian coordinates	4<br>Energy = -335.8508407745<br>Rh -0.0000000 0.0000000 0.0000000<br>O 0.8378061 -1.4511227 0.0000000<br>O 0.8378061 1.4511227 0.0000000<br>O -1.6756121 0.0000000 0.0000000
Vibrational data	# mode symmetry wave number IR intensity selection rules<br># cm**(-1) km/mol IR RAMAN<br>7 a2" 45.64 5.32851 YES NO<br>8 e' 278.61 0.35281 YES YES<br>9 e' 278.61 0.35281 YES YES<br>10 e' 915.01 34.98778 YES YES<br>11 e' 915.01 34.98778 YES YES<br>12 a1' 926.68 0.00000 NO YES
**RHF/UCCSD(T)/aug-cc-pVTZ(-PP) (C_{2v} – $^1A_1$)**	
Cartesian coordinates	4<br>UCCSD(T)/AUG-CC-PVTZ,RH=AUG-CC-PVTZ-PP ENERGY=-334.61603634<br>Rh 0.0000000000 0.0000000000 -0.0000000000<br>O 0.0000000000 0.0000000000 -1.6945422520<br>O 0.0000000000 -1.4675166380 0.8472711260<br>O 0.0000000000 1.4675166380 0.8472711260

Table S7. Optimized structures, point groups used for calculations and vibrational data for the T_1 electronic state.

**B3LYP/def2-TZVP (C_{2v} - $^3A_2$)**	
Cartesian coordinates	4<br>Energy = -335.6622806982<br>Rh 0.0000000 0.0000000 -0.0509802<br>O 0.0000000 1.4369856 0.9084276<br>O 0.0000000 -1.4369856 0.9084276<br>O 0.0000000 0.0000000 -1.7658750
Vibrational data	# mode symmetry wave number IR intensity selection rules<br># cm**(-1) km/mol IR RAMAN<br>7 b1 175.66 15.49553 YES YES<br>8 b2 214.70 1.08030 YES YES<br>9 a1 263.95 0.64144 YES YES<br>10 b2 682.80 6.69940 YES YES<br>11 a1 739.50 11.23338 YES YES<br>12 a1 768.80 4.58906 YES YES
**M06L/def2-TZVP (C_{2v} - $^3A_2$)**	
Cartesian coordinates	4<br>Energy = -335.8473093563<br>Rh 0.0000000 0.0000000 -0.0604150<br>O 0.0000000 1.4203087 0.9160031<br>O 0.0000000 -1.4203087 0.9160031<br>O 0.0000000 0.0000000 -1.7715912
Vibrational data	# mode symmetry wave number IR intensity selection rules<br># cm**(-1) km/mol IR RAMAN<br>7 b1 160.27 16.01832 YES YES<br>8 b2 197.96 1.13187 YES YES

	9 a1 277.40 0.24854 YES YES<br>10 b2 710.82 10.36639 YES YES<br>11 a1 745.84 6.75616 YES YES<br>12 a1 804.68 15.66957 YES YES
**ROHF-UCCSD(T)/aug-cc-pVTZ(-PP) (C_{2v} - $^3A_2$)**	
Cartesian coordinates	4<br>UCCSD(T)/AUG-CC-PVTZ,RH=AUG-CC-PVTZ-PP ENERGY=-334.58887572<br>Rh 0.0000000000 0.0000000000 0.0120054561<br>O 0.0000000000 0.0000000000 1.7604541673<br>O 0.0000000000 1.4354471900 -0.9188356398<br>O 0.0000000000 -1.4354471900 -0.9188356398

Table S8. Optimized structures, point groups used for calculations and vibrational data for the T_2 electronic state.

**B3LYP/def2-TZVP (C_s - $^3A'$)**	
Cartesian coordinates	4<br>Energy = -335.6534362692<br>Rh -0.2806835 -0.0933182 0.0000000<br>O 0.1072163 -0.7639758 1.4919944<br>O 0.1072163 -0.7639758 -1.4919944<br>O 0.0662509 1.6212699 0.0000000
Vibrational data	# mode symmetry wave number IR intensity selection rules<br># cm**(-1) km/mol IR RAMAN<br>7 a' 220.36 8.12959 YES YES<br>8 a' 270.79 1.50722 YES YES<br>9 a" 313.96 0.16221 YES YES<br>10 a' 686.16 1.03030 YES YES<br>11 a' 862.75 1.95507 YES YES<br>12 a" 1033.38 24.55692 YES YES
**M06L/def2-TZVP (C_s - $^3A'$)**	
Cartesian coordinates	4<br>Energy = -335.8422650738<br>Rh -0.2621840 -0.1040540 0.0000000<br>O 0.0551403 1.6161201 0.0000000<br>O 0.1035219 -0.7560331 -1.5060489<br>O 0.1035219 -0.7560331 1.5060489
Vibrational data	# mode symmetry wave number IR intensity selection rules<br># cm**(-1) km/mol IR RAMAN<br>7 a' 202.35 9.29295 YES YES<br>8 a' 271.57 1.11387 YES YES<br>9 a" 301.11 8.91067 YES YES<br>10 a' 714.42 0.96359 YES YES<br>11 a' 878.18 5.22054 YES YES<br>12 a" 904.92 0.39949 YES YES
**ROHF-UCCSD(T)/aug-cc-pVTZ(-PP) (C_{2v} - $^3B_1$)**	
Cartesian coordinates	4<br>UCCSD(T)/AUG-CC-PVTZ,RH=AUG-CC-PVTZ-PP ENERGY=-334.59230784<br>Rh 0.0000000000 0.0000000000 -0.0434403590<br>O 0.0000000000 0.0000000000 1.7023322818<br>O 0.0000000000 1.5595770613 -0.7114655315<br>O 0.0000000000 -1.5595770613 -0.7114655315

Table S9. Optimized structures, point groups used for calculations and vibrational data for the T_3 electronic state.

**B3LYP/def2-TZVP (C_{2v} - $^3B_2$)**	
Cartesian coordinates	4<br>Energy = -335.5969451132<br>Rh 0.0000000 0.0000000 0.0000301<br>O 0.0000000 1.4790265 0.8538636<br>O 0.0000000 -1.4790265 0.8538636<br>O 0.0000000 0.0000000 -1.7077574
Vibrational data	# mode symmetry wave number IR intensity selection rules<br># cm**(-1) km/mol IR RAMAN<br>7 b2 268.57 0.04553 YES YES<br>8 a1 268.68 0.04575 YES YES<br>9 b1 304.02 12.52429 YES YES<br>10 a1 773.35 7.92149 YES YES<br>11 b2 773.38 7.92300 YES YES<br>12 a1 853.26 0.00000 YES YES

**M06L/def2-TZVP (C_{2v} - $^3B_2$)**	
Cartesian coordinates	4<br>Energy = -335.7816295267<br>Rh 0.0000000 0.0000000 0.0001973<br>O 0.0000000 1.4833074 0.8560654<br>O 0.0000000 -1.4833074 0.8560654<br>O 0.0000000 0.0000000 -1.7123281
Vibrational data	# mode symmetry wave number IR intensity selection rules<br># cm**(-1) km/mol IR RAMAN<br>7 a1 266.13 0.17120 YES YES<br>8 b2 267.47 0.14718 YES YES<br>9 b1 284.98 14.17339 YES YES<br>10 a1 798.95 23.52586 YES YES<br>11 b2 799.27 24.53594 YES YES<br>12 a1 832.81 0.04377 YES YES
**ROHF-UCCSD(T)/aug-cc-pVTZ(-PP) (C_{2v} - $^3B_2$)**	
Cartesian coordinates	4<br>UCCSD(T)/AUG-CC-PVTZ,RH=AUG-CC-PVTZ-PP ENERGY=-334.53190761<br>Rh 0.0000000000 0.0000000000 0.0000093553<br>O 0.0000000000 1.4855563213 0.8576702096<br>O 0.0000000000 -1.4855563213 0.8576702096<br>O 0.0000000000 0.0000000000 -1.7154005908

Table S10. Optimized structures, point groups used for calculations and vibrational data for the Q_1 electronic state.

**B3LYP/def2-TZVP (D_{3h} - $^5A''_1$)**	
Cartesian coordinates	4<br>Energy = -335.6432828530<br>Rh 0.0000000 0.0000000 0.0000000<br>O 0.8787980 -1.5221229 0.0000000<br>O 0.8787980 1.5221229 0.0000000<br>O -1.7575961 0.0000000 0.0000000
Vibrational data	# mode symmetry wave number IR intensity selection rules<br># cm**(-1) km/mol IR RAMAN<br>7 e' 222.82 0.99504 YES YES<br>8 e' 222.82 0.99504 YES YES<br>9 a2" 266.02 8.22439 YES NO<br>10 e' 624.56 8.10380 YES YES<br>11 e' 624.56 8.10380 YES YES<br>12 a1' 780.39 0.00000 NO YES
**M06L/def2-TZVP (D_{3h} - $^5A''_1$)**	
Cartesian coordinates	4<br>Energy = -335.8221914194<br>Rh -0.0000000 0.0000000 0.0000000<br>O 0.8798965 -1.5240254 0.0000000<br>O 0.8798965 1.5240254 0.0000000<br>O -1.7597929 0.0000000 0.0000000
Vibrational data	# mode symmetry wave number IR intensity selection rules<br># cm**(-1) km/mol IR RAMAN<br>7 e' 213.37 1.07270 YES YES<br>8 e' 213.37 1.07270 YES YES<br>9 a2" 257.40 8.10988 YES NO<br>10 e' 607.93 3.27347 YES YES<br>11 e' 607.93 3.27347 YES YES<br>12 a1' 776.43 0.00000 NO YES
**ROHF-UCCSD(T)/aug-cc-pVTZ(-PP) (C_{2v} - $^5A_2$)**	
Cartesian coordinates	4<br>UCCSD(T)/AUG-CC-PVTZ,RH=AUG-CC-PVTZ-PP ENERGY=-334.55745014<br>Rh 0.0000000000 0.0000000000 -0.0000000000<br>O 0.0000000000 0.0000000000 -1.7599810954<br>O 0.0000000000 -1.5241883388 0.8799905477<br>O 0.0000000000 1.5241883388 0.8799905477

References

[1] A. Wijaikhum et al. "Absolute ozone densities in a radio-frequency driven atmospheric pressure plasma using two-beam UV-LED absorption spectroscopy and numerical simulations". In: *Plasma Sources Science and Technology* 26.11 (2017), p. 115004.

[2] Manfred M. Kappes and Ralph H. Staley. "Oxidation of transition-metal cations in the gas phase. Oxygen bond dissociation energies and formation of an excited-state product". In: *The Journal of Physical Chemistry* 85.8 (1981), pp. 942–944.

[3] S. Feil et al. "Ozone reactions with alkaline-earth metal cations and dications in the gas phase: Room-temperature kinetics and catalysis". In: *Journal of Physical Chemistry A* 111.51 (2007), pp. 13397–13402.

[4] Joshua J. Melko et al. "Determining Rate Constants and Mechanisms for Sequential Reactions of Fe^+ with Ozone at 500 K". In: *Journal of Physical Chemistry A* 121.1 (2017), pp. 24–30.

[5] Mickaël G Delcey et al. "Soft X-ray signatures of cationic manganese–oxo systems, including a high-spin manganese(v) complex". In: *Physical Chemistry Chemical Physics* 24.6 (2022), pp. 3598–3610.

[6] S. H. Vosko, L. Wilk, and M. Nusair. "Accurate spin-dependent electron liquid correlation energies for local spin density calculations: a critical analysis". In: *Canadian Journal of Physics* 58.8 (1980), pp. 1200–1211.

[7] Chengteh Lee, Weitao Yang, and Robert G. Parr. "Development of the Colle-Salvetti correlation-energy formula into a functional of the electron density". In: *Physical Review B* 37.2 (Jan. 1988), pp. 785–789.

[8] Axel D. Becke. "A new mixing of Hartree-Fock and local density-functional theories". In: *The Journal of Chemical Physics* 98.2 (1993), pp. 1372–1377.

[9] P J Stephen et al. "Ab Initio Calculation of Vibrational Absorption". In: *The Journal of Physical Chemistry* 98.45 (1994), pp. 11623–11627.

[10] Yan Zhao and Donald G. Truhlar. "The M06 suite of density functionals for main group thermochemistry, thermochemical kinetics, noncovalent interactions, excited states, and transition elements: Two new functionals and systematic testing of four M06-class functionals and 12 other functionals". In: *Theoretical Chemistry Accounts* 120.1-3 (2008), pp. 215–241.

[11] Yan Zhao and Donald G. Truhlar. "A new local density functional for main-group thermochemistry, transition metal bonding, thermochemical kinetics, and noncovalent interactions". In: *Journal of Chemical Physics* 125.19 (2006).

[12] D. Andrae et al. "Energy-adjusted ab initio pseudopotentials for the second and third row transition elements". In: *Theoretica Chimica Acta* 77.2 (1990), pp. 123–141.

[13] Florian Weigend et al. "RI-MP2: Optimized auxiliary basis sets and demonstration of efficiency". In: *Chemical Physics Letters* 294.1-3 (1998), pp. 143–152.

[14] Florian Weigend and Reinhart Ahlrichs. "Balanced basis sets of split valence, triple zeta valence and quadruple zeta valence quality for H to Rn: Design and assessment of accuracy". In: *Physical Chemistry Chemical Physics* 7.18 (2005), pp. 3297–3305.

[15] *TURBOMOLE V7.2 2017, a development of University of Karlsruhe and Forschungszentrum Karlsruhe GmbH, 1989-2007, TURBOMOLE GmbH, since 2007; available from* `http://www.turbomole.com`.

[16] Kirk A. Peterson et al. "Energy-consistent relativistic pseudopotentials and correlation consistent basis sets for the 4d elements Y-Pd". In: *Journal of Chemical Physics* 126.12 (2007).

[17] Rick A. Kendall, Thom H. Dunning, and Robert J. Harrison. "Electron affinities of the first-row atoms revisited. Systematic basis sets and wave functions". In: *The Journal of Chemical Physics* 96.9 (1992), pp. 6796–6806.

[18] Hans Joachim Werner et al. "Molpro: A general-purpose quantum chemistry program package". In: *Wiley Interdisciplinary Reviews: Computational Molecular Science* 2.2 (2012), pp. 242–253.

[19] H.-J. Werner et al. *MOLPRO, version , a package of ab initio programs*. Stuttgart, Germany, 2019.

[20] Hans Joachim Werner et al. "The Molpro quantum chemistry package". In: *Journal of Chemical Physics* 152.14 (2020).

[21] John P. Perdew. "Erratum: Density-functional approximation for the correlation energy of the inhomogeneous electron gas (Physical Review B (1986) 34, 10 (7406))". In: *Physical Review B* 34.10 (1986), p. 7406.

[22] Florian Weigend. "Accurate Coulomb-fitting basis sets for H to Rn". In: *Physical Chemistry Chemical Physics* 8.9 (2006), pp. 1057–1065.

[23] Julian D. Rolfes, Frank Neese, and Dimitrios A. Pantazis. "All-electron scalar relativistic basis sets for the elements Rb–Xe". In: *Journal of Computational Chemistry* 41.20 (2020), pp. 1842–1849.

[24] Frank Neese. "Software update: the ORCA program system, version 4.0". In: *Wiley Interdisciplinary Reviews: Computational Molecular Science* 8.1 (2018), pp. 4–9.

[25] Serena DeBeer George and Frank Neese. "Calibration of Scalar Relativistic Density Functional Theory for the Calculation of Sulfur K-Edge X-ray Absorption Spectra". In: *Inorganic Chemistry* 49.4 (Feb. 2010), pp. 1849–1853.

[26] Kallol Ray et al. "Description of the ground-state covalencies of the bis(dithiolato) transition-metal Complexes from X-ray absorption spectroscopy and time-dependent density-functional calculations". In: *Chemistry - A European Journal* 13.10 (2007), pp. 2783–2797.

[27] Giovanni Ghigo, Björn O. Roos, and Per-Åke Malmqvist. "A Modified Definition of the Zeroth-Order Hamiltonian in Multiconfigurational Perturbation Theory (CASPT2)". In: *Chemical Physics Letters* 396.1 (2004), pp. 142–149.

[28] Kerstin Andersson and Björn O. Roos. "Multiconfigurational Second-Order Perturbation Theory: A Test of Geometries and Binding Energies". In: *International Journal of Quantum Chemistry* 45.6 (1993), pp. 591–607.

[29] J. Patrick Zobel, Juan J. Nogueira, and Leticia González. "The IPEA Dilemma in CASPT2". In: *Chemical Science* 8.2 (2017), pp. 1482–1499.
